# Human FGF-21 Is a Substrate of Fibroblast Activation Protein

**DOI:** 10.1371/journal.pone.0151269

**Published:** 2016-03-10

**Authors:** Andrew L. Coppage, Kathryn R. Heard, Matthew T. DiMare, Yuxin Liu, Wengen Wu, Jack H. Lai, William W. Bachovchin

**Affiliations:** 1 Department of Developmental, Molecular and Chemical Biology, Tufts University Sackler School of Graduate Biomedical Sciences, Boston, Massachusetts, United States of America; 2 Arisaph Pharmaceuticals Inc., Boston, Massachusetts, United States of America; University of Barcelona, Faculty of Biology, SPAIN

## Abstract

FGF-21 is a key regulator of metabolism and potential drug candidate for the treatment of type II diabetes and other metabolic disorders. However, the half-life of active, circulating, human FGF-21 has recently been shown to be limited in mice and monkeys by a proteolytic cleavage between P171 and S172. Here, we show that fibroblast activation protein is the enzyme responsible for this proteolysis by demonstrating that purified FAP cleaves human FGF-21 at this site *in vitro*, and that an FAP-specific inhibitor, ARI-3099, blocks the activity in mouse, monkey and human plasma and prolongs the half-life of circulating human FGF-21 in mice. Mouse FGF-21, however, lacks the FAP cleavage site and is not cleaved by FAP. These findings indicate FAP may function in the regulation of metabolism and that FAP inhibitors may prove useful in the treatment of diabetes and metabolic disorders in humans, but pre-clinical proof of concept studies in rodents will be problematic.

## Introduction

Fibroblast Growth Factor 21 (FGF-21, Q9NSA1), a member of the FGF super-family of proteins, plays a role in the regulation of glucose and lipid metabolism [1–3]. Knockout of the FGF-21 gene in mice leads to a mild increase in weight gain and reduced glucose tolerance [4]. Whereas mice engineered to over express FGF-21 are lean, and have improved glucose tolerance [5].

Consistent with the genetic studies, pharmacological administration of FGF-21 improves both glucose tolerance and insulin sensitivity in ob/ob and diet-induced obese mice, reduces triglycerides, LDL and total cholesterol in db/db mice, and improves glucose and lipid homeostasis in diabetic rhesus monkeys without inducing hypoglycemia [6–10]. The anti-diabetic activities observed in animal models also appear to apply to humans, as the FGF-21 analog LY2405319 improves the lipoprotein profile and lowers glucose in obese patients with type II diabetes [11].

FGF-21 exerts its effects on metabolism by acting as a hormone capable of signaling multiple cell types [12,13]. FGF-21 is primarily expressed by the liver, where it can act in an autocrine fashion to activate FGF signaling and reduce hepatic glucose output [14]. In addition, FGF-21 strongly stimulates glucose uptake in adipocytes by binding fibroblast growth factor receptors (FGFRs) and beta-klotho (bKlotho) on the cell surface [15]. Receptor activation leads to translocation of glucose transporter 1 (GLUT1) and cellular glucose uptake [16]. FGF-21 also appears to target the pancreas, where it preserves and improves beta cell function [17]. Other effects of FGF-21 include enhanced fatty acid oxidation, reduced hepatic triglyceride synthesis through inhibition of sterol regulatory element-binding protein 1 (SREBP-1) and increased metabolic rate [18–20].

The ability of FGF-21 to positively impact glucose and lipid homeostasis in the context of metabolic syndrome has led to investigation of FGF-21 as a therapeutic molecule for type 2 diabetes. However, injection of recombinant FGF-21 may be of limited clinical value due to a half-life of only 1–2 hours in animal models [1]. Reportedly contributing to this limited half-life is a proteolytic event by an unknown enzyme between P171 and S172 of human FGF-21 when injected into mice and monkeys [21]. Previous work has demonstrated that FGF-21 truncated at this site exhibits a 400-fold reduction in the ability to activate FGF signaling in adipocytes [22]. This suggests that proteolysis of human FGF-21 at this site may be functionally consequential.

Primary sequence analysis of human FGF-21 indicated the presence of a glycine in P2 and a proline in the P1 position, which is a consensus site for the endopeptidase activity of the extracellular post-proline cleaving enzyme fibroblast activation protein (FAP) [23–25]. In fact, FAP has recently been implicated in the regulation of metabolism due to the diabetes and obesity-resistant phenotype of the FAP knockout mouse [26]. While the mouse form of FGF-21 lacks this FAP cut site, the mouse phenotype nonetheless suggests that FAP may function in the regulation of metabolically active peptides and proteins and that cleavage of FGF-21 by FAP in humans may be biologically significant. Therefore, we investigated FGF-21 as a substrate of FAP.

## Materials and Methods

### *In vitro* FGF-21 digests

Recombinant human FGF-21 (Peprotech) or recombinant mouse FGF-21 (ProSpec Protein Specialists) was reconstituted in FAP assay buffer (50 mM Tris, 140 mM NaCl, pH 7.5). Reactions were carried out at a final concentration of 20 μM FGF-21, 200 nM recombinant human FAP (R&D systems) or PREP (R&D systems) and 16 μM ARI-3099. For SDS-PAGE analysis, samples were immediately added to 2x gel loading buffer (0.6 ml 1M Tris pH 6.8, 2.5 ml 50% glycerol, 2 ml 10% SDS, 1 ml 1% bromophenol blue, 3.4 ml H20 and 0.25 ml β-mercaptoethanol/ 5.5 ml aliquot). 3 μg of protein was then loaded onto a reducing 20% SDS-PAGE gel. Gels were stained with Gelcode Blue Stain Reagent (Thermo Scientific). Alternatively, for LC/MS, aliquots of the reaction were taken and quenched with 10% v/v .01 M HCL and run on 1100 series LC/MSD (Agilent and HP). LC solvents were H_2_O+.01% TFA (solvent A) and acetonitrile+.08% TFA (solvent B). LC was set to 2% solvent B 0–2 minutes followed by 40–88% solvent B gradient from 2–30 minutes (Column: Zorbax C-18, 2.2 x 50 mm, 3.5 μM). Percent cleavage of FGF-21 was quantified by extracted ion chromatogram integration of peaks corresponding to the +10, +11 and +12 ions of both cleaved and intact FGF-21. The half-life was calculated using one phase decay function on GraphPad Prism software.

### Intact FGF-21 ELISA validation

Recombinant human FGF-21 was reconstituted in FAP assay buffer. FGF-21 at 20 μM was incubated with or without 500 nM recombinant human FAP. Reactions were incubated at 37°C for 5 hours and then serially diluted in FAP assay buffer. Levels of intact human FGF-21 from these reactions were assessed by Human Intact Fibroblast Growth Factor ELISA (Eagle Biosciences, according to the manufacturer’s instructions). FGF-21 digested by FAP was not recognized by this ELISA.

### Plasma FGF-21 digests

Pooled human or cynomolgus monkey plasma (Innovative Research) or pooled mouse plasma from C57BL/6J mice (Jackson Laboratory) was incubated with recombinant human FGF-21 in FAP assay buffer with or without ARI-3099. Final concentrations were 1 μM for FGF-21 and 16 μM for ARI-3099. Reactions were incubated at 37°C for 24 hours and levels of intact FGF-21 were assessed by Human Intact Fibroblast Growth Factor ELISA.

### Plasma FAP activity measurements

In triplicate, plasma samples were diluted in PBS to 1 mg/ml and 180 μl of diluted sample was added to a 96 well plate followed by 20 μl of 500 μM ARI-3144 substrate solution. Data was collected by a spectromax M2^e^ fluorescent plate reader (Molecular Devices) over 30 minutes at 37°C (ex. 380, em. 460).

### Pharmacodynamics of FAP inhibition with ARI-3099 in mouse

C57BL/6J mice were administered ARI-3099 at 80 mg/kg in a PBS vehicle via oral gavage. Blood samples were collected by tail vein nick before and after compound administration at the indicated time points and plasma was immediately isolated by centrifugation. FAP activity was assessed using ARI-3144 as described above.

### Clearance and degradation of human FGF-21 in mouse

80 mg/kg ARI-3099 or PBS vehicle was administered to C57BL/6J mice subcutaneously, followed 1 hour later by I.P. injection of human FGF-21 at 0.5 mg/kg in PBS. Blood samples were collected by tail vein nick and plasma was immediately isolated by centrifugation. Levels of intact FGF-21 were assessed by Human Intact Fibroblast Growth Factor ELISA.

### Ethics statement

All experiments were carried out in accordance with the protocol B2011-29 approved by the Tufts University Institutional Animal Care and Use Committee (IACUC).

## Results

### FAP cleaves human FGF-21 after Pro-171

**Fig 1:**
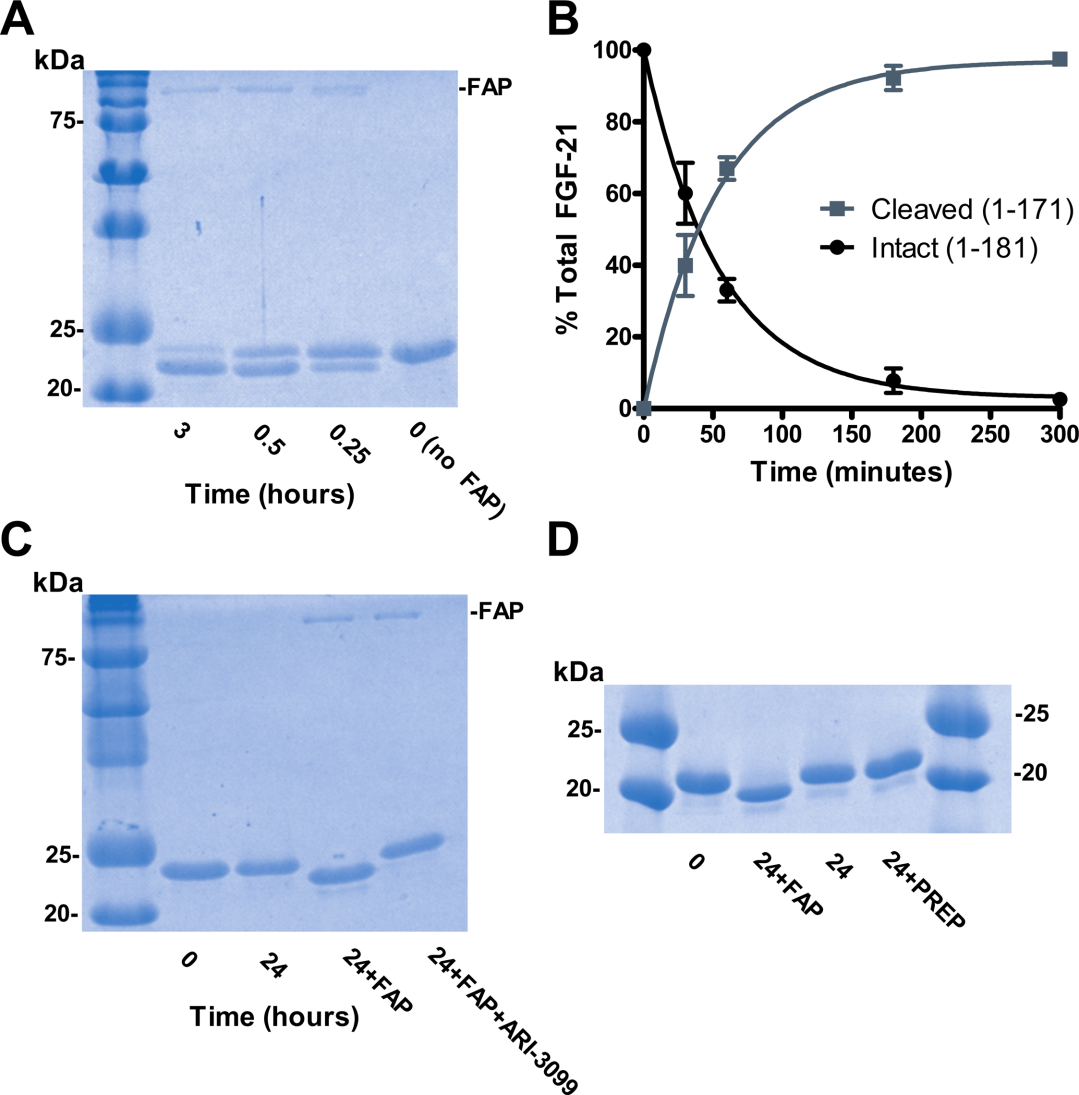
Human FGF-21 is digested by FAP but not PREP. (A) Human FGF-21 is cleaved by FAP. Recombinant human FGF-21 was digested by recombinant human FAP and visualized by Coomassie staining of SDS-Page gel. (B) Time course of FGF-21 digestion by FAP quantified by LC/MS extracted ion integration of peaks corresponding to intact (1–181) and cleaved (1–171) forms of FGF-21 (n = 3 per time point per group). Values are mean ± SEM with one phase decay curve fit superimposed. (C) FAP cleavage of FGF-21 is prevented by ARI-3099. ARI-3099 was pre-incubated with recombinant FAP for 30 minutes prior to addition of FGF-21. Reaction products were visualized by Coomassie staining of SDS-Page gel. (D) Recombinant PREP does not cleave FGF-21. Recombinant human PREP was added to recombinant FGF-21 and visualized by Coomassie staining of SDS-Page gel.

To test our hypothesis, we first determined if FAP could cleave recombinant human FGF-21 *in vitro*. FGF-21 incubated with recombinant human FAP was analyzed by SDS-PAGE (Fig 1A). Over the course of three hours, the FGF-21 band decreased and a new band appeared with an apparent MW consistent with the predicted fragment of FGF-21 (1–171). LC/MS analysis confirmed the appearance the FGF-21 (1–171) and (172–181) fragments and the disappearance of intact FGF-21 with a half-life of approximately 38 minutes (Fig 1B). These were the only two detectable fragments resulting from FAP cleavage. Additionally, proteolysis of FGF-21 by FAP was completely inhibited by the FAP-specific competitive inhibitor ARI-3099 (Fig 1C) [27]. Prolyl endopeptidase (PREP), though closely related to FAP and also capable of post-prolyl endopeptidase activity, failed to cleave FGF-21 even after 24 hours of incubation (Fig 1D) [24].

In contrast, mouse FGF-21 (Q9JJN1), which has a glutamic acid instead of glycine at the P2 site, is not cleaved by FAP (S1 Fig). Furthermore, mutation of the P2 residue in human FGF-21 from G to E is reported to abolish proteolysis at this site [21]. Both of these findings are consistent with FAP mediating the observed cleavage because FAP is known to require glycine at P2 for endoproteolytic cleavage activity before a proline at P1 [23–25].

### Human FGF-21 is cleaved by FAP in plasma

A soluble FAP is known to be present in blood plasma with levels being higher in mice than in humans [28]. To assess the effects of plasma derived FAP on FGF-21 degradation, recombinant human FGF-21 was added to mouse, cynomolgus monkey and human plasma. Levels of intact FGF-21 were assessed by sandwich ELISA, with antibodies directed to the N and C-termini of FGF-21. The C-terminal antibody targets residues removed by FAP cleavage and FAP-cleaved FGF-21 is not recognized by this ELISA (S2 Fig).

**Fig 2:**
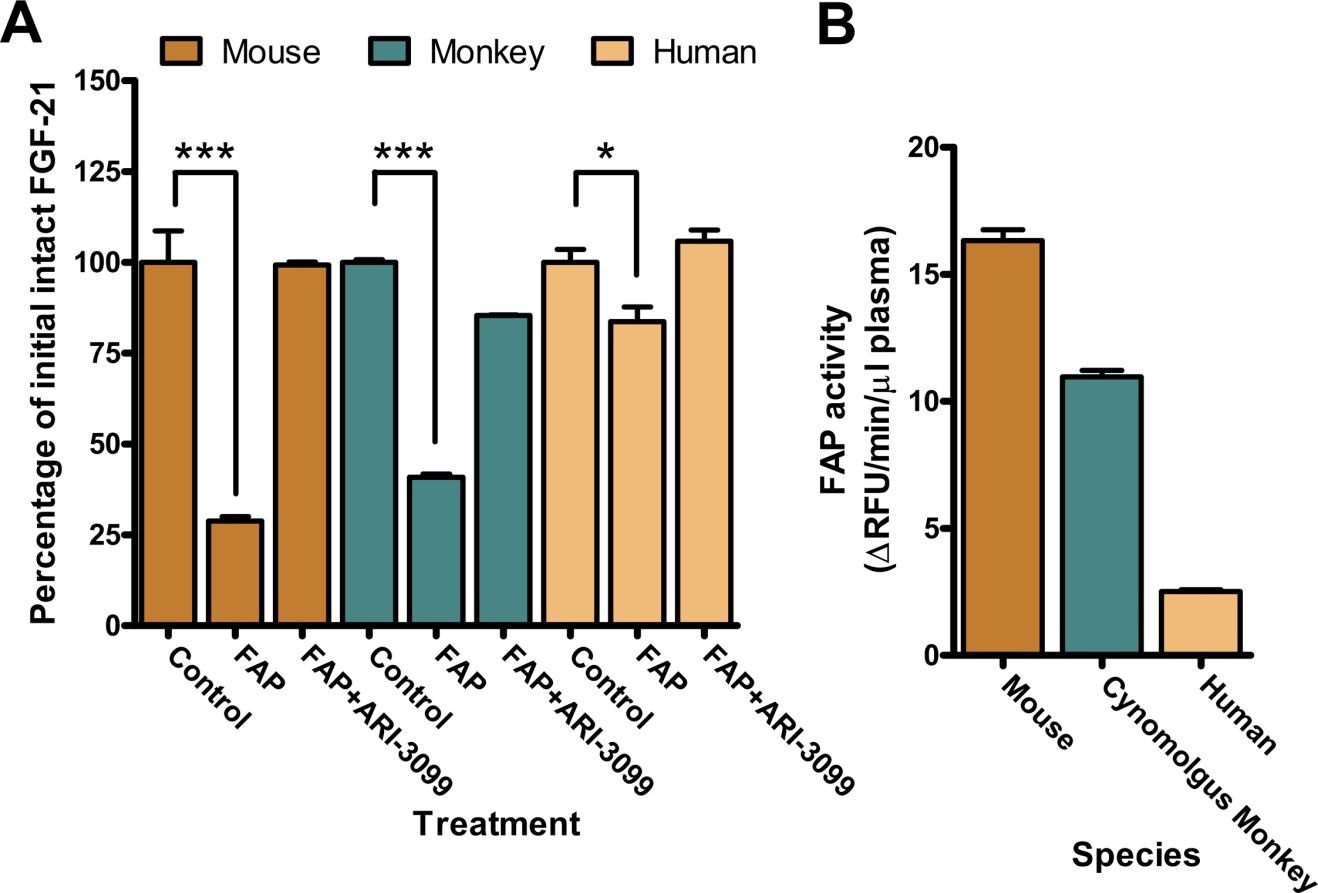
Effect of FAP inhibition on FGF-21 digestion in plasma. (A) FAP cleaves human FGF-21 in mouse, monkey and human plasma. Recombinant FGF-21 was added to plasma to a final concentration of 1 μM in the presence or absence of 16 μM ARI-3099 followed by assessment of intact FGF-21 by sandwich ELISA (n = 3 per group). Values are mean ± SEM. *P < .05 ***P < .001 by *ANOVA*. (B) FAP activity of mouse, monkey and human plasma as assessed by the FAP-specific fluorescent substrate ARI-3144.

Human FGF-21 incubated in mouse and monkey plasma was degraded over time confirming earlier reports (Fig 2A) [21]. Human plasma was also found to degrade FGF-21, albeit to a lesser degree. Loss of intact FGF-21 was greatest in mouse plasma, followed by monkey, and least in human plasma, correlating well with the relative amounts of FAP activity in plasma from each species as assessed by the FAP-specific fluorescent substrate ARI-3144 (Fig 2B) [28]. Addition of the FAP-specific inhibitor ARI-3099 prevented FGF-21 degradation, proving that FAP is the enzyme in the plasma from each species mediating the cleavage.

### Inhibition of FAP extends the half-life of intact human FGF-21 in mouse

**Fig 3:**
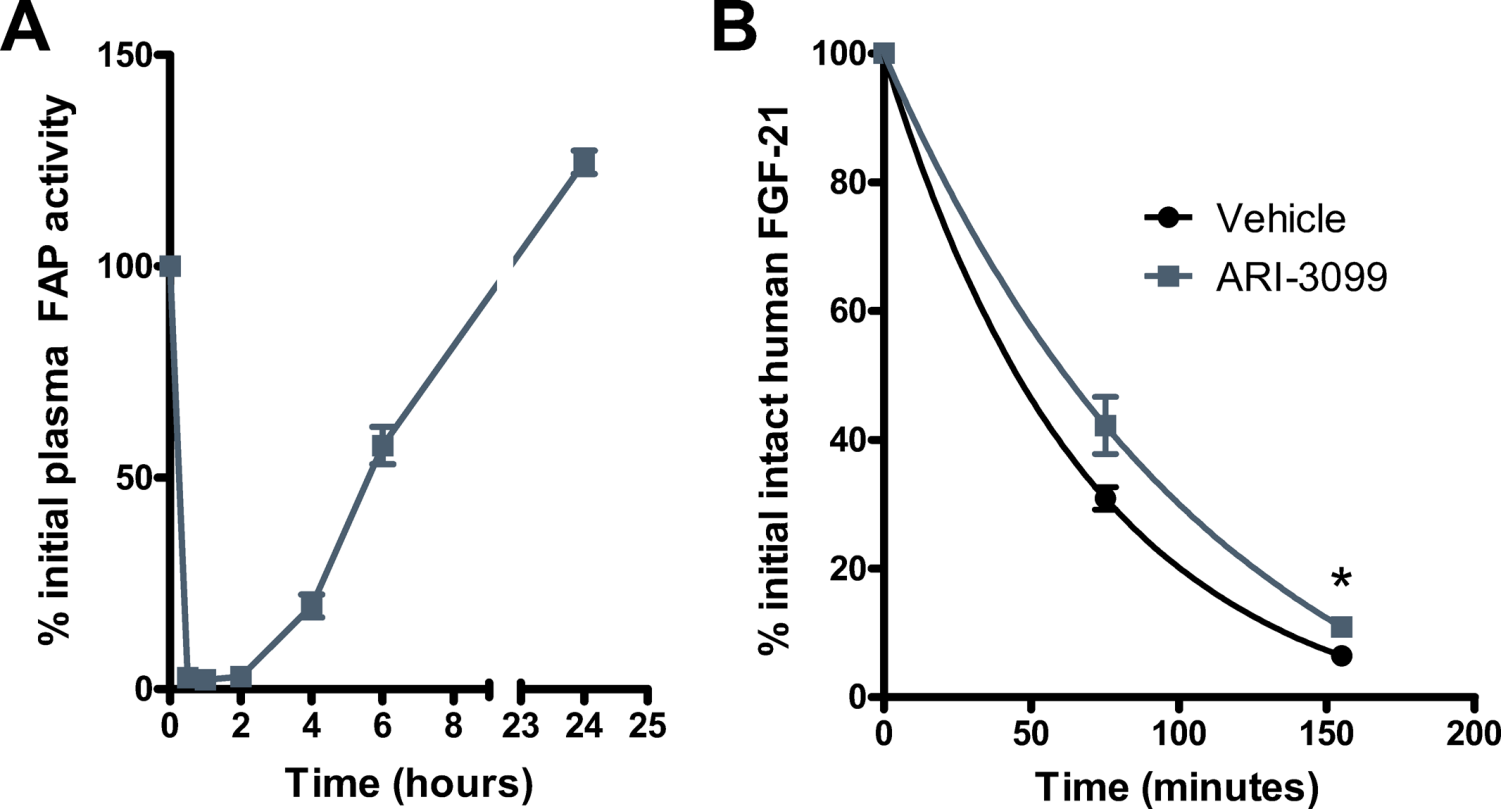
Inhibition of FAP prolongs the half-life of human FGF-21 in mice. (A) ARI-3099 was administered to mice at 80 mg/kg via oral gavage. FAP activity in plasma samples was determined using the FAP-specific fluorescent substrate ARI-3144 (n = 3 per group). Values are mean ± SEM. (B) Mice were pre-treated with vehicle or 80 mg/kg ARI-3099 followed by injection of 0.5 mg/kg human FGF-21 (n = 4 per group). Plasma samples were assessed for intact FGF-21 concentrations by sandwich ELISA. Values are mean ± SEM. *P < .05 by *t*-test.

We next examined FGF-21 administration in conjunction with FAP inhibition in mice. Renal filtration is reported to be the primary mode of FGF-21 clearance [1,29]. However, proteolysis is reported to contribute to the elimination of human FGF-21, at least at pharmacological doses [21]. To determine whether the contribution from proteolysis is mediated by FAP, we administered mice ARI-3099 followed by recombinant human FGF-21. At the chosen dose of 80 mg/kg, ARI-3099 potently suppresses plasma FAP activity as measured by ARI-3144 (Fig 3A). Pre-treatment with this dose of ARI-3099 increased the amount of intact FGF-21 detected in the plasma and extended its estimated half-life from 48 to 79 min (Fig 3B). These results suggest that FAP-mediated cleavage contributes to shortening the half-life of human FGF-21 *in vivo* in mice.

## Discussion

Our data clearly demonstrate the ability of FAP to cleave human FGF-21 between P171 and S172. This cleavage site is consistent with the reported requirements for FAP endopeptidase cleavage with glycine in P2 position and proline in P1. Though this site is also a consensus sequence for PREP endopeptidase activity, recombinant PREP is unable to cleave FGF-21. This is likely due to PREP’s preference for small peptide substrates with fewer than 30 amino acids. FGF-21, at nearly 20kDa, may be too large to be a PREP substrate. In contrast, FAP has the ability to cleave larger proteins [23]. FGF-21 also has another GP motif at residues 132–133 that could be subject to recognition and cleavage by FAP [24]. However, the predicted structure of FGF-21 is a single globular domain flanked by disordered N and C termini. This Gly-Pro motif lies within the globular domain of FGF-21 and is therefore not likely to be accessible for proteolysis.

The N-terminus of human FGF-21 also contains both penultimate and preantepenultimate proline residues that may also be susceptible to the dipeptidyl peptidase activity of FAP or related proteases. Indeed, other work has suggested these residues may be subject to proteolysis by amino-peptidases or dipeptidyl-peptidases [30]. However, the N-terminal methionine of the recombinant FGF-21 utilized in this work and most others prevents evaluating these sites for susceptibility to dipeptidyl peptidase cleavage. Even if proteolysis occurs at these sites *in vivo*, receptor signaling capability does not appear to be impaired by removal of N-terminal residues 1–4 of FGF-21 suggesting truncation at these sites, may be functionally inconsequential [31].

Unlike these other sites, FAP cleavage of human FGF-21 is likely to result in a significant loss of function because the C-terminus of FGF-21 is critical for its ability to activate downstream signaling pathways. The C-terminal region of FGF-21 binds the co-receptor bKlotho while the N-terminal region binds FGFRs on the cell surface [22]. FGF-21 lacking the 10 C-terminal residues, which is produced upon FAP cleavage, exhibits impaired binding to bKlotho and this truncated FGF-21 is 400-fold less potent in a reporter assay designed to reflect the degree of extracellular signal-regulated kinase (ERK) phosphorylation in cultured adipocytes [31].

While this article was being prepared, two separate groups reported the ability of FAP to cleave 10 amino acids off the C-terminus of human FGF-21 [32,33]. Additionally, these studies utilized a form of FGF-21 without an N-terminal methionine. Using this recombinant FGF-21, these groups were able to confirm the ability of FAP to cleave after the proline-2 and proline-4 N-terminal residues.

While FAP cleavage of human FGF-21 produces a truncated protein that is impaired in its signaling capabilities, the biological significance of this proteolysis *in vivo* remains to be determined. Our data, both in plasma and mice, strengthens a previous assertion that FAP proteolysis limits the half-life of pharmacological doses of human FGF-21 [21]. However, due to limitations of our sandwich ELISA assay, we cannot specifically detect levels of FAP-cleaved FGF-21. Therefore, it is possible, albeit unlikely that our FAP-specific inhibitor affects clearance of FGF-21 as opposed to proteolysis when injected into mice.

Also of particular interest is the degree to which FAP proteolysis may alter FGF-21 signaling at pharmacological or physiological concentrations, which includes the ability of FGF-21 to alter ERK phosphorylation in adipose tissue and drive glucose uptake. However, mouse FGF-21 lacks the FAP cut site and varying FAP activity levels and rates of kidney clearance in pre-clinical models may provide a poor proxy for assessing the signaling consequences of FAP cleavage of native FGF-21 in humans. Furthermore, although our data demonstrated minimal, albeit statistically significant, cleavage of FGF-21 in human plasma, this result was obtained at far higher concentrations of FGF-21 (1 μM) than would be observed physiologically. Therefore, this result not entirely representative of the degree to which FAP cleavage of FGF-21 might be observed in humans, especially when one also considers the contribution of the membrane bound enzyme. The recently published results from Dunshee et. al demonstrate that pharmacological inhibition of FAP increases endogenous levels of intact FGF-21 in cynomolgus monkeys, further suggesting that the interaction of FAP and FGF-21 is likely to be relevant in *vivo* [33].

The present results indicate that FGF-21 may be a biologically relevant substrate for FAP in humans, but not in mice and therefore, that FAP inhibitors may prove useful in the treatment of diabetes and metabolic disorders in humans by increasing the lifetime of FGF-21. Unfortunately, the fact that mouse FGF-21 is not susceptible to the FAP-mediated cleavage makes preclinical proof of concept studies as well as other preclinical studies regarding the effects of pharmacological blockade of FAP-mediated processing of FGF-21 problematic. However, that the observation that FAP knockout mice are resistant to diet-induced obesity and the metabolic abnormalities associated with obesity indicates that other FAP substrates may be involved in regulating metabolism in mice and remain to be discovered [26]. Therefore, FAP inhibitors could prove even more useful in treating diabetes and metabolic disorders than may be expected from extending the lifetime of FGF-21 alone. Further work aimed at identifying the biological substrates and function of FAP is needed and likely to be consequential.

## Supporting Information

S1 Fig Mouse FGF-21 is not cleaved by FAP. Recombinant mouse FGF-21 was digested by recombinant human FAP and visualized by Coomassie staining of SDS-Page gel.
(TIFF)

S2 Fig Intact FGF-21 sandwich ELISA does not recognize FAP-cleaved FGF-21. 20 μM recombinant human FGF-21 was incubated with or without 500 nM recombinant human FAP at 37°C for 5 hours. Reactions were then diluted into the range of the standard curve and assayed for intact FGF-21 using the intact FGF-21 sandwich ELISA.
(TIFF)

## References

[1] Kharitonenkov A, Shiyanova TL, Koester A, Ford AM, Micanovic R, Galbreath EJ, et al FGF-21 as a novel metabolic regulator. J Clin Invest. 2005 6;115(6):1627–35. 15902306 10.1172/JCI23606 PMC1088017

[2] Long YC, Kharitonenkov A. Hormone-like fibroblast growth factors and metabolic regulation. Biochim Biophys Acta. 2011 7;1812(7):791–5. 10.1016/j.bbadis.2011.04.002 21504790

[3] Popovici C, Roubin R, Coulier F, Birnbaum D. An evolutionary history of the FGF superfamily. Bioessays. 2005 8;27(8):849–57. 16015590 10.1002/bies.20261

[4] Badman MK, Koester A, Flier JS, Kharitonenkov A, Maratos-Flier E. Fibroblast growth factor 21-deficient mice demonstrate impaired adaptation to ketosis. Endocrinology. 2009 11;150(11):4931–40. 10.1210/en.2009-0532 19819944 PMC2775979

[5] Hotta Y, Nakamura H, Konishi M, Murata Y, Takagi H, Matsumura S, et al Fibroblast growth factor 21 regulates lipolysis in white adipose tissue but is not required for ketogenesis and triglyceride clearance in liver. Endocrinology. 2009 10;150(10):4625–33. 10.1210/en.2009-0119 19589869

[6] Xu J, Stanislaus S, Chinookoswong N, Lau YY, Hager T, Patel J, et al Acute glucose-lowering and insulin-sensitizing action of FGF21 in insulin-resistant mouse models—association with liver and adipose tissue effects. Am J Physiol Endocrinol Metab. 2009 11;297(5):E1105–14. 10.1152/ajpendo.00348.2009 19706786

[7] Kim HW, Lee JE, Cha JJ, Hyun YY, Kim JE, Lee MH, et al Fibroblast growth factor 21 improves insulin resistance and ameliorates renal injury in db/db mice. Endocrinology. 2013 9;154(9):3366–76. 10.1210/en.2012-2276 23825123

[8] Veniant MM, Komorowski R, Chen P, Stanislaus S, Winters K, Hager T, et al Long-acting FGF21 has enhanced efficacy in diet-induced obese mice and in obese rhesus monkeys. Endocrinology. 2012 9;153(9):4192–203. 10.1210/en.2012-1211 22798348

[9] Kharitonenkov A, Wroblewski VJ, Koester A, Chen YF, Clutinger CK, Tigno XT, et al The metabolic state of diabetic monkeys is regulated by fibroblast growth factor-21. Endocrinology. 2007 2;148(2):774–81. 17068132 10.1210/en.2006-1168

[10] Fisher FM, Chui PC, Antonellis PJ, Bina HA, Kharitonenkov A, Flier JS, et al Obesity is a fibroblast growth factor 21 (FGF21)-resistant state. Diabetes. 2010 11;59(11):2781–9. 10.2337/db10-0193 20682689 PMC2963536

[11] Gaich G, Chien JY, Fu H, Glass LC, Deeg MA, Holland WL, et al The effects of LY2405319, an FGF21 analog, in obese human subjects with type 2 diabetes. Cell Metab. 2013 9 3;18(3):333–40. 10.1016/j.cmet.2013.08.005 24011069

[12] Adams AC, Coskun T, Rovira AR, Schneider MA, Raches DW, Micanovic R, et al Fundamentals of FGF19 & FGF21 action in vitro and in vivo. PLoS One. 2012;7(5):e38438 10.1371/journal.pone.0038438 22675463 PMC3365001

[13] Zhang X, Yeung DC, Karpisek M, Stejskal D, Zhou ZG, Liu F, et al Serum FGF21 levels are increased in obesity and are independently associated with the metabolic syndrome in humans. Diabetes. 2008 5;57(5):1246–53. 10.2337/db07-1476 18252893

[14] Nishimura T, Nakatake Y, Konishi M, Itoh N. Identification of a novel FGF, FGF-21, preferentially expressed in the liver. Biochim Biophys Acta. 2000 6 21;1492(1):203–6. 10858549 10.1016/s0167-4781(00)00067-1

[15] Goetz R, Beenken A, Ibrahimi OA, Kalinina J, Olsen SK, Eliseenkova AV, et al Molecular insights into the klotho-dependent, endocrine mode of action of fibroblast growth factor 19 subfamily members. Mol Cell Biol. 2007 5;27(9):3417–28. 17339340 10.1128/MCB.02249-06 PMC1899957

[16] Ge X, Chen C, Hui X, Wang Y, Lam KS, Xu A. Fibroblast growth factor 21 induces glucose transporter-1 expression through activation of the serum response factor/Ets-like protein-1 in adipocytes. J Biol Chem. 2011 10 7;286(40):34533–41. 10.1074/jbc.M111.248591 21846717 PMC3186365

[17] Wente W, Efanov AM, Brenner M, Kharitonenkov A, Koster A, Sandusky GE, et al Fibroblast growth factor-21 improves pancreatic beta-cell function and survival by activation of extracellular signal-regulated kinase 1/2 and Akt signaling pathways. Diabetes. 2006 9;55(9):2470–8. 16936195 10.2337/db05-1435

[18] Zhang Y, Xie Y, Berglund ED, Coate KC, He TT, Katafuchi T, et al The starvation hormone, fibroblast growth factor-21, extends lifespan in mice. Elife. 2012;1:e00065 10.7554/eLife.00065 23066506 PMC3466591

[19] Zhang Y, Lei T, Huang JF, Wang SB, Zhou LL, Yang ZQ, et al The link between fibroblast growth factor 21 and sterol regulatory element binding protein 1c during lipogenesis in hepatocytes. Mol Cell Endocrinol. 2011 8 6;342(1–2):41–7. 10.1016/j.mce.2011.05.003 21664250

[20] Xu J, Lloyd DJ, Hale C, Stanislaus S, Chen M, Sivits G, et al Fibroblast growth factor 21 reverses hepatic steatosis, increases energy expenditure, and improves insulin sensitivity in diet-induced obese mice. Diabetes. 2009 1;58(1):250–9. 10.2337/db08-0392 18840786 PMC2606881

[21] Hecht R, Li YS, Sun J, Belouski E, Hall M, Hager T, et al Rationale-Based Engineering of a Potent Long-Acting FGF21 Analog for the Treatment of Type 2 Diabetes. PLoS One. 2012;7(11):e49345 10.1371/journal.pone.0049345 23209571 PMC3507880

[22] Micanovic R, Raches DW, Dunbar JD, Driver DA, Bina HA, Dickinson CD, et al Different roles of N- and C- termini in the functional activity of FGF21. J Cell Physiol. 2009 5;219(2):227–34. 10.1002/jcp.21675 19117008

[23] Huang CH, Suen CS, Lin CT, Chien CH, Lee HY, Chung KM, et al Cleavage-site specificity of prolyl endopeptidase FAP investigated with a full-length protein substrate. J Biochem. 2011 6;149(6):685–92. 10.1093/jb/mvr017 21288888

[24] Jambunathan K, Watson DS, Endsley AN, Kodukula K, Galande AK. Comparative analysis of the substrate preferences of two post-proline cleaving endopeptidases, prolyl oligopeptidase and fibroblast activation protein alpha. FEBS Lett. 2012 7 30;586(16):2507–12. 10.1016/j.febslet.2012.06.015 22750443 PMC3500622

[25] Edosada CY, Quan C, Wiesmann C, Tran T, Sutherlin D, Reynolds M, et al Selective inhibition of fibroblast activation protein protease based on dipeptide substrate specificity. J Biol Chem. 2006 3 17;281(11):7437–44. 16410248 10.1074/jbc.M511112200

[26] Gorrell M, Sunmi S, Wang X, Novel Metabolic Disease Therapy. United States. 2012.

[27] Poplawski SE, Lai JH, Li Y, Jin Z, Liu Y, Wu W, et al Identification of selective and potent inhibitors of fibroblast activation protein and prolyl oligopeptidase. J Med Chem. 2013 5 9;56(9):3467–77. 10.1021/jm400351a 23594271 PMC4059180

[28] Keane FM, Yao TW, Seelk S, Gall MG, Chowdhury S, Poplawski SE, et al Quantitation of fibroblast activation protein (FAP)-specific protease activity in mouse, baboon and human fluids and organs. FEBS Open Bio. 2013;4:43–54. 10.1016/j.fob.2013.12.001 24371721 PMC3871272

[29] Stein S, Bachmann A, Lossner U, Kratzsch J, Bluher M, Stumvoll M, et al Serum levels of the adipokine FGF21 depend on renal function. Diabetes Care. 2009 1;32(1):126–8. 10.2337/dc08-1054 18840768 PMC2606845

[30] Kharitonenkov A, Beals JM, Micanovic R, Strifler BA, Rathnachalam R, Wroblewski VJ, et al Rational design of a fibroblast growth factor 21-based clinical candidate, LY2405319. PLoS One. 2013;8(3):e58575 10.1371/journal.pone.0058575 23536797 PMC3594191

[31] Yie J, Hecht R, Patel J, Stevens J, Wang W, Hawkins N, et al FGF21 N- and C-termini play different roles in receptor interaction and activation. FEBS Lett. 2009 1 5;583(1):19–24. 10.1016/j.febslet.2008.11.023 19059246

[32] Zhen EY, Jin Z, Ackermann BL, Thomas MK, Gutierrez JA. Circulating FGF21 Proteolytic Processing Mediated by Fibroblast Activation Protein. Biochem J. 2015 12 3. 10.1042/BJ20151085 PMC4764976 26635356

[33] Dunshee DR, Bainbridge TW, Kljavin NM, Zavala-Solorio J, Schroeder AC, Chan R, et al Fibroblast Activation Protein Cleaves and Inactivates Fibroblast Growth Factor 21. J Biol Chem. 2016 1 21. 10.1074/jbc.M115.710582 PMC4786731 26797127

