# Supplementary figures and images for: Human FGF-21 Is a Substrate of Fibroblast Activation Protein
Source: PLoS One. 2016 Mar 10;11(3):e0151269. doi: 10.1371/journal.pone.0151269 (PMC4786124; doi:10.1371/journal.pone.0151269)

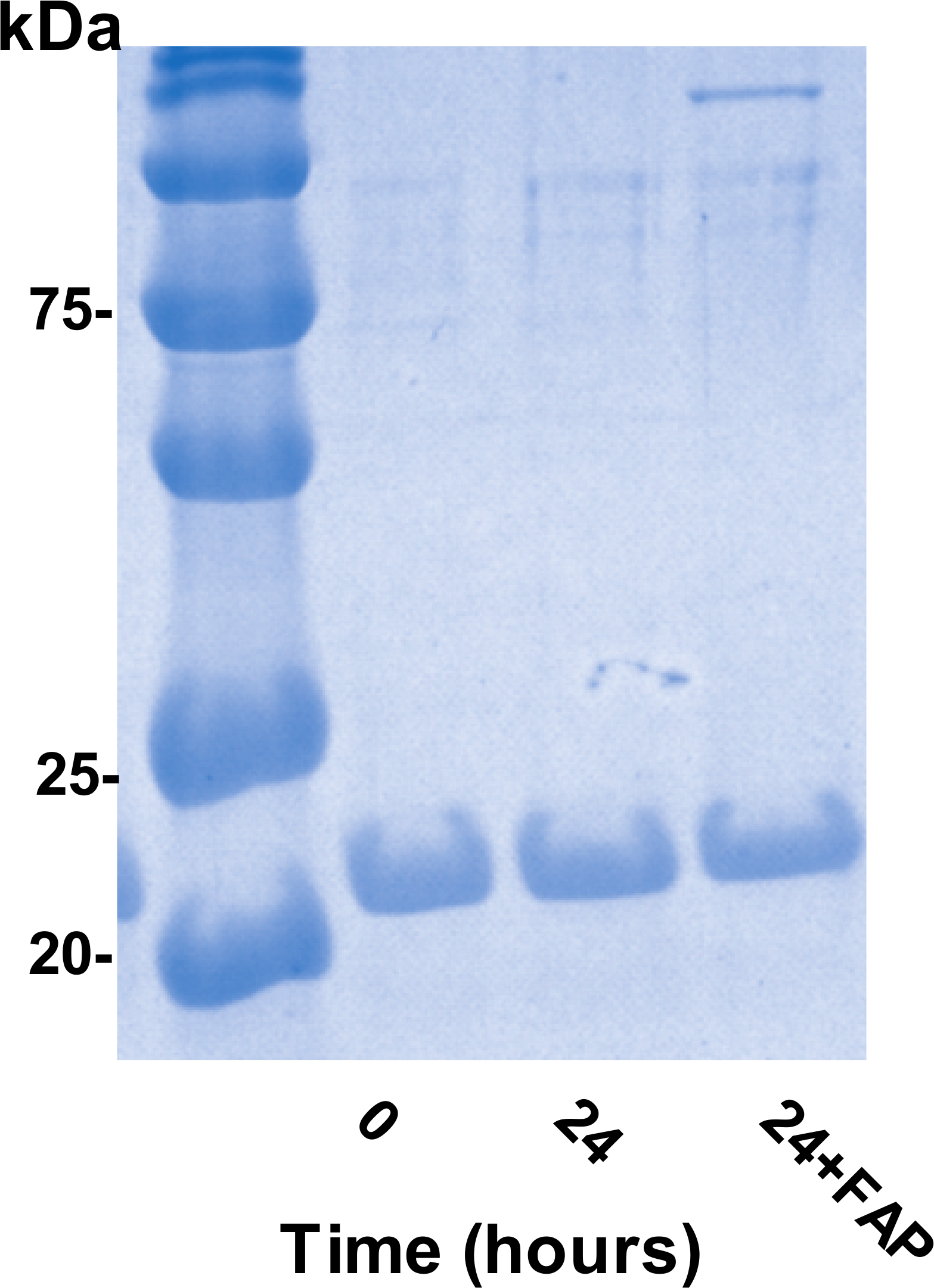

Supplement: S1 Fig — Recombinant mouse FGF-21 was digested by recombinant human FAP and visualized by Coomassie staining of SDS-Page gel. (TIFF) [file pone.0151269.s001.tiff]

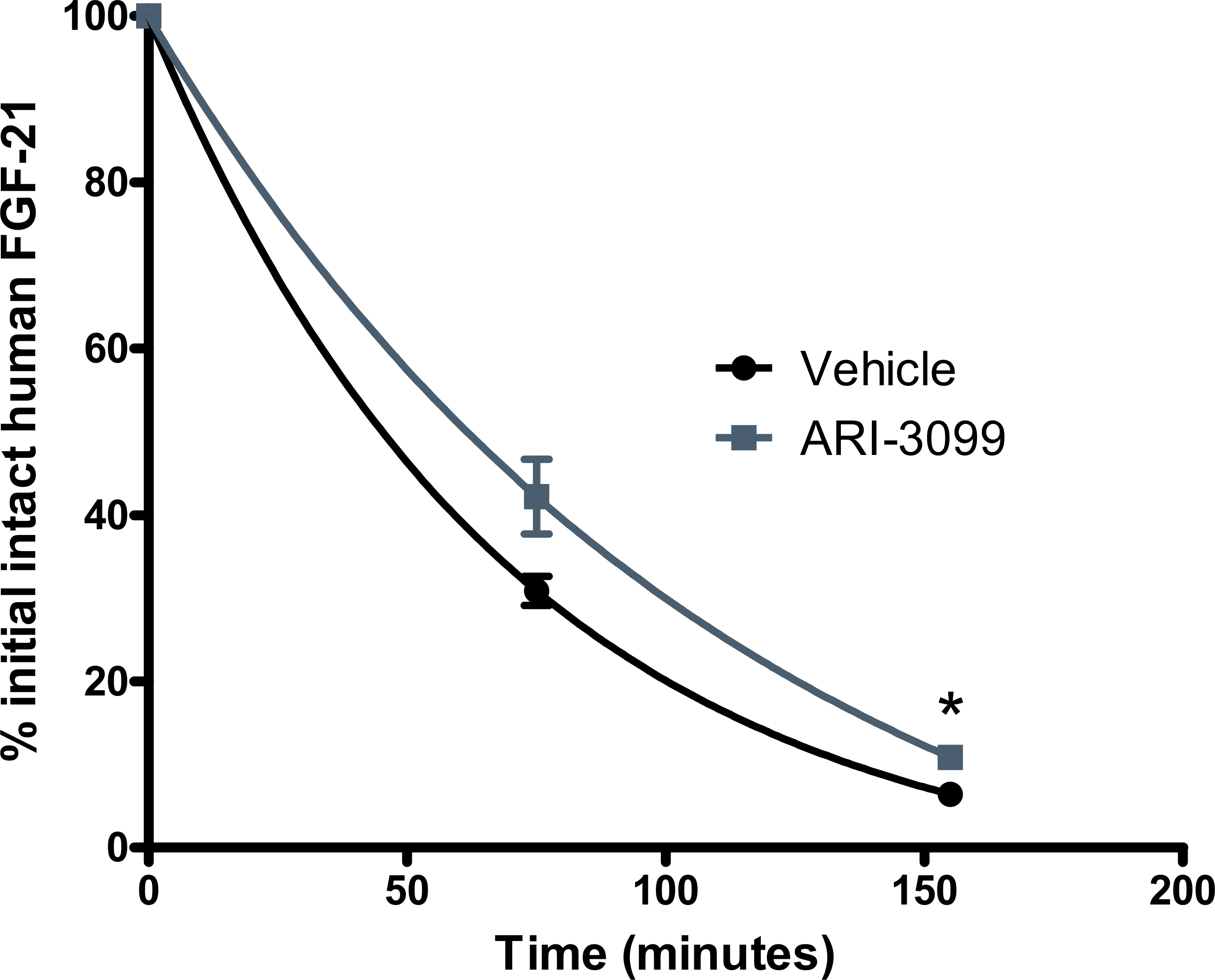

Supplement: S2 Fig — 20 μM recombinant human FGF-21 was incubated with or without 500 nM recombinant human FAP at 37°C for 5 hours. Reactions were then diluted into the range of the standard curve and assayed for intact FGF-21 using the intact FGF-21 sandwich ELISA. (TIFF) [file pone.0151269.s002.tiff]
